# Energy Detection Based on Undecimated Discrete Wavelet Transform and Its Application in Magnetic Anomaly Detection

**DOI:** 10.1371/journal.pone.0110829

**Published:** 2014-10-24

**Authors:** Xinhua Nie, Zhongming Pan, Dasha Zhang, Han Zhou, Min Chen, Wenna Zhang

**Affiliations:** College of Mechatronics Engineering and Automation, National University of Defense Technology, Changsha, Hunan, P. R. China; Xiamen University, China

## Abstract

Magnetic anomaly detection (MAD) is a passive approach for detection of a ferromagnetic target, and its performance is often limited by external noises. In consideration of one major noise source is the fractal noise (or called 1/*f* noise) with a power spectral density of 1/*f^a^* (0<*a*<2), which is non-stationary, self-similarity and long-range correlation. Meanwhile the orthonormal wavelet decomposition can play the role of a Karhunen-Loève-type expansion to the 1/*f*-type signal by its decorrelation abilities, an effective energy detection method based on undecimated discrete wavelet transform (UDWT) is proposed in this paper. Firstly, the foundations of magnetic anomaly detection and UDWT are introduced in brief, while a possible detection system based on giant magneto-impedance (GMI) magnetic sensor is also given out. Then our proposed energy detection based on UDWT is described in detail, and the probabilities of false alarm and detection for given the detection threshold in theory are presented. It is noticeable that no a priori assumptions regarding the ferromagnetic target or the magnetic noise probability are necessary for our method, and different from the discrete wavelet transform (DWT), the UDWT is shift invariant. Finally, some simulations are performed and the results show that the detection performance of our proposed detector is better than that of the conventional energy detector even utilized in the Gaussian white noise, especially when the spectral parameter *α* is less than 1.0. In addition, a real-world experiment was done to demonstrate the advantages of the proposed method.

## Introduction

Magnetic anomaly detection (MAD) is a kind of magnetic technology for detecting, localizing, and tracking the visually obscured ferromagnetic targets [1]–[5], such as vehicles, unexploded ordnances (UXO), and wrecks of sunken ships and submarines, etc. Farther away from the target, the magnetic field produced by the target can be modeled as a dipole field. Thus the total measured magnetic field ***B*** *_M_* can be expressed as: 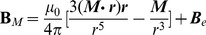
(1)where ***B*** *_e_* is the geomagnetic field, ***M*** denotes the target magnetic moment, ***r*** is the vector from the target to the measurement point (magnetic sensor), and *μ*_0_ = 4π×10^−7^ T.m/A is the permeability of free space. Then the principle of magnetic anomaly detection is detecting the anomaly appears in the geomagnetic field ***B*** *_e_*.

Aire Sheinker and Boris Ginzburg, et al, divided MAD methods into two major categories: target-based method and noise-based method [2]. The fore category is based on analyzing target signal typical patterns [3], [4], and several assumptions regarding the target are usually required, such as: (1) the target can be represented by a single magnetic dipole model; (2) the target moves along a straight line passing by the magnetic sensor; and (3) the target characteristic time *τ* is a known priori. The later one reveals changes in the magnetic background nature, which is assumed that the changes are caused by the presence of a ferromagnetic target [2], [5]. It would be adaptive to the magnetic background as usual, and no a priori assumptions regarding the target are required, which may result in a simpler implementation and lower power consumption [2], [5].

In fact, the practical measured magnetic field ***B*** *_M_* is usually contaminated by the external noise, such as magnetic noise and electronic device noise. And one of the major noise sources is the 1/*f* fractal noise (or called 1/*f* noise) with a power spectral density of 1/*f^α^* (here, *α* is the spectral parameter, and 0<*α*<2) [4], which is non-stationary, self-similarity, and long-range correlation. Thus the performances of the traditional detectors, for example, the energy detector, designed for the case of the target signal contaminated by Gaussian white noise, cannot be effectively handled [4]. In addition, a high-order crossing approach was used to detect the visually obscured ferromagnetic objects by revealing the anomalies in the ambient geomagnetic field ***B_e_*** [2]. And a noise-based MAD method based on adaptive minimum entropy detector was proposed to detect any changes in the magnetic noise pattern [5]. However, both these two methods mostly rely on a priori magnetic noise probability density function. In this paper, a novel energy detection method based on undecimated discrete wavelet transform (UDWT), which can effectively remove the self-similarity and long-range correlation of the 1/*f* noise [6], is proposed. Especially, no a priori assumptions regarding the target or the magnetic noise probability are necessary for our proposed method. Furthermore, different from the discrete wavelet transform (DWT), the undecimated wavelet transform is shift invariant, and then the performance of the detector is also shift invariant. Moreover, the length of decomposed coefficient is changeless in the UDWT case.

The rest of this article is organized as follows: In Section 2, some basic concepts of magnetic anomaly detection and UDWT are introduced in brief, while one possible detection circuit based on giant magneto-impedance (GMI) magnetic sensor are also given out. The principles of our proposed energy detector based on UDWT are described in detail in Section 3. Some performances of our detector are illustrated in Section 4, while our conclusions and prospects are presented in Section 5.

## Principles

### 2.1 Magnetic target signal model

**Figure 1:**
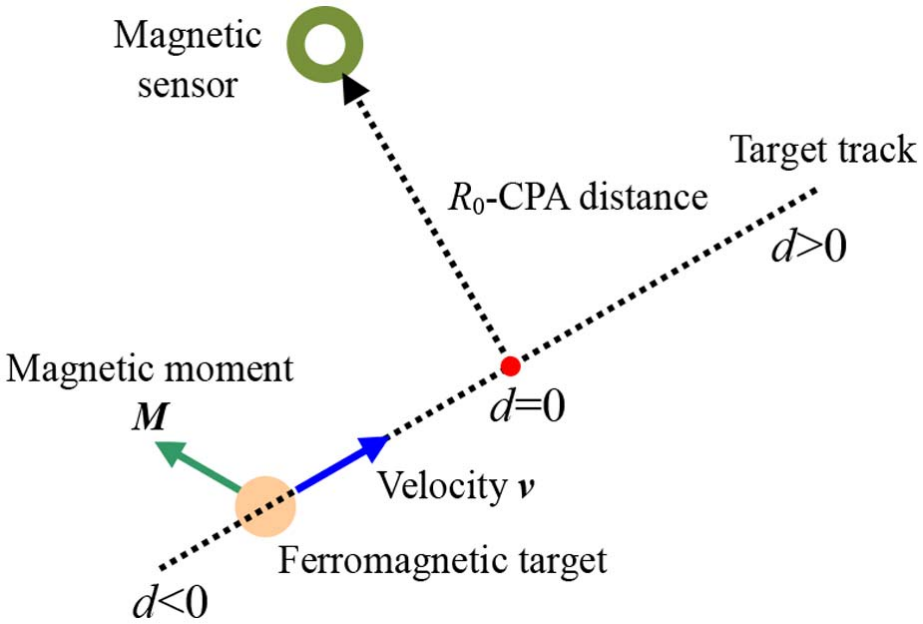
Diagram of a static magnetic sensor place to detect a ferromagnetic target moving along a straight line.

The case of a static magnetic sensor sensing a moving ferromagnetic target is illustrated in Fig. 1. Of course, the case that the ferromagnetic target is static while the magnetic sensor moves is in the similar way [4]. In order to model the target signal, several assumptions regarding the target are presented: (1) The target moves along a straight line track with a constant velocity ***v***; (2) The target magnetic moment ***M*** is constant in magnitude and orientation.

Using the Gram-Schmidt procedure, the target signal *s*(*n*) can be represented as a linear combination of three orthonormal basis functions (OBFs) *f_k_*(*n*) (*k* = 1, 2, 3) defined as follow [2]:
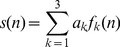
(2)where
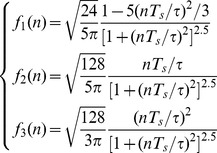
(3)here, the characteristic time *τ* is defined by the ratio *τ = R*_0_ */v*, where *R*_0_ is the closet proximity approach (CPA) distance and *v* is the velocity of the target moving along the straight track. *T_s_* denotes the sampling period. In reference [2], authors gave some conclusions that: (1) The larger values of *τ* correspond to wider target signals; (2) Most of the target signal energy is concentrated at low frequencies. However, neither the velocity *v* nor CPA distance *R*_0_ is known a priori. Thus the characteristic time *τ* should be estimated, which would bring some errors into the target model. In Ref. [3], [4], authors adopt a multi-channel approach, in which each channel is associated with a different characteristic time *τ*, and detection occurs whenever one or more channel outputs rise above the given threshold.

### 2.2 Undecimated discrete wavelet transform (UDWT)

The wavelet transform is a signal processing technique that represents a transient or non-stationary signal in terms of time and scale distribution, which is an excellent tool for on-line data compression, analysis and reducing, etc. [7], [8]. In this section, we review some basic concepts and definitions of undecimated wavelet transform that are important in the content of this paper.

**Figure 2:**
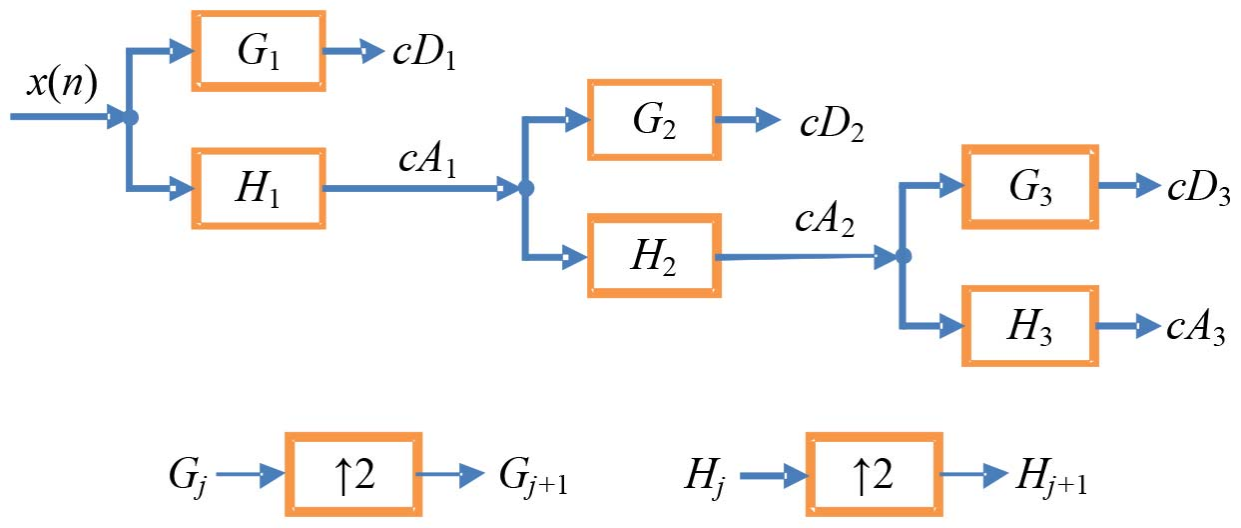
Three-level decomposition algorithm based on UDWT.

As an example, Fig. 2 shows the scheme of the three-level decomposition algorithm based on undecimated wavelet transform, which is known as a two-channel sub-band filter. *G_j_*(*k*) and *H_j_*(*k*) are the decomposition high-pass and low-pass filters, respectively. While the symbol ↑2 denotes up-sampling by 2.

As illustrated in Fig. 2, the time-domain signal *x*(*n*) is passed through a series of high-pass filters *G_j_*(*k*) to analyze the high frequencies, referred to as detail coefficients *cD_j_* at *j*th level. Synchronously, the signal *x*(*n*) is also passed through a series of low-pass filters *H_j_*(*k*) in order to analyze its low frequencies, called approximation coefficients *cA_j_*. Here, the high-pass filters *G_j_*(*k*) and low-pass filters *H_j_*(*k*) constitute ‘quadrature mirror filters’ and exactly half-band filters.

### 2.3 Detection circuit

**Figure 3:**
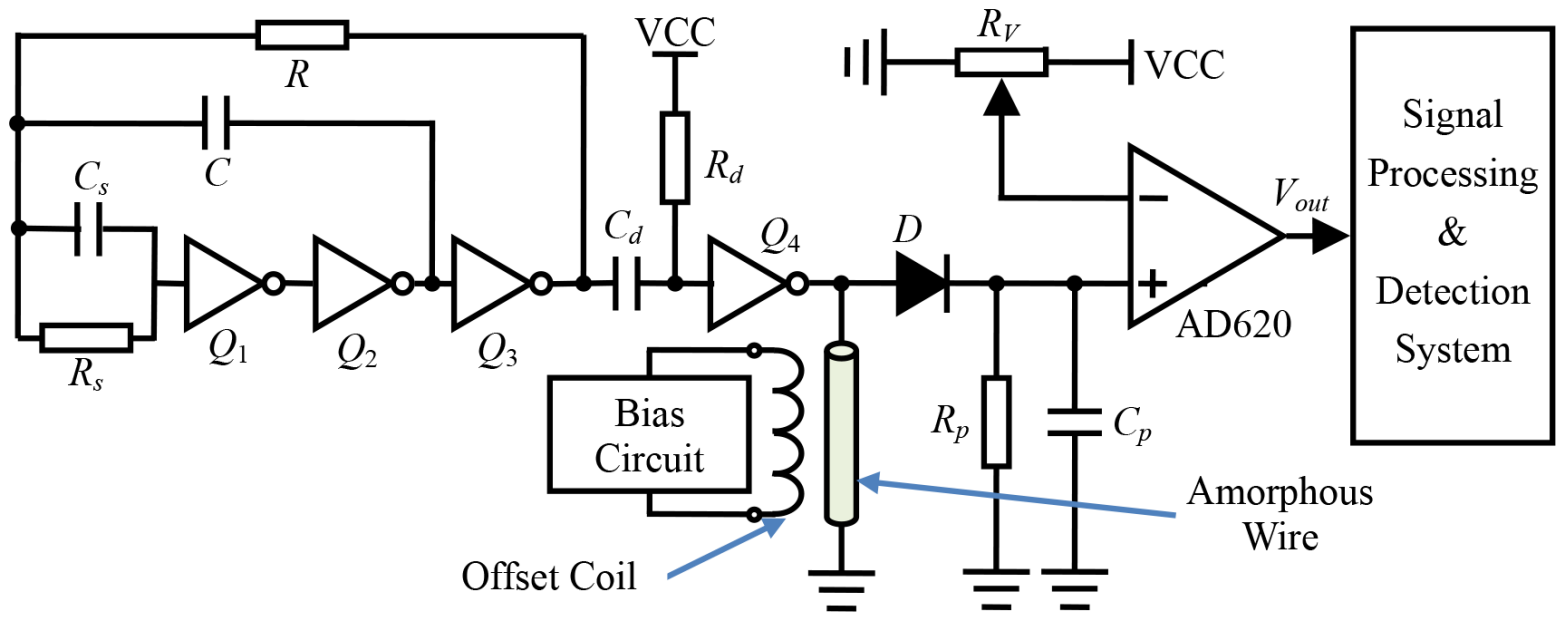
The conditioning circuit of the GMI magnetic sensor.

Recently, magnetic sensors have been extensively studied for many years because of their potential applications in nearly all engineering and industrial sectors, such as navigation, target detection and tracking, etc. [9], [10]. And the development of high performance magnetic sensors has benefited from the discovery of the giant magneto-impedance (GMI) effect which is well known as a magnetic phenomenon that a large change in the impedance (*Z*) of a ferromagnetic conductor (ribbon- or wire-shaped) with a small alternating current (*I*) can be achieved upon applying an external magnetic field (*H_ex_*) tangential to the length of the conductor [11]–[13]. Here, one possible conditioning circuit of the GMI magnetic sensor based on peak-detecting technology is illustrated in Fig. 3.

As illustrated in Fig. 3, an asymmetrical multivibrator is composed of CMOS inverters *Q*_1_∼*Q*_3_, *R_s_*, *C_s_*, *R* and *C*, and the period of its output square voltage is about *T*≈2.2×*RC*. Here, *R_s_* and *C_s_* are low resistance and capacitor, respectively, which can effectively restrain the input current of the CMOS inverters, and make the output voltage waveform to be more stable. In addition, a differential circuit (*R_d_* and *C_d_*) then reformed through a CMOS inverter *Q*_4_ to apply a sharp pulse train to the amorphous wire [14].

On the other hand, the amorphous wire usually does not have a bipolar response near-zero magnetic. To shift the operation point to the linear part of the characteristic, a bias magnetic field *H_dc_* is generated by the basis circuit and the offset coil winding around the amorphous wire, and its strength can be adjusted by controlling the DC current provided by the bias circuit [15], [16].

The response signal induced in the amorphous wire due to the GMI effect are developed by the external magnetic field *H_ex_*, which is then converted to DC voltage through a peak detector composed by the Schottky diode *D*, *R_p_*, and *C_p_*. After passing the low-passing filter (*R_p_* and *C_p_*), the output voltage *V_out_* is generated through a differential instrumentation amplifier AD620 with zero adjustment, and then connected to the input of signal processing and detection system.

Multiple measurements show that our GMI magnetic sensor exhibits a linearity error about 0.98%FS in the measuring range of ±2.0 Oe, and its sensitivity can achieve about 748 mV/Oe. Besides, the bandwidth of our sensor is more than 2.0 kHz at -3 dB can be observed, and its average noise power spectral density is about 1.3 nT/Hz^1/2^. In other words, as the measurement bandwidth of our GMI magnetic sensor is 2.0 kHz, its corresponding magnetic field resolution is 58 nT. For its good performance, this GMI magnetic sensor can be utilized to detecting the magnetic anomaly signal appears in the weak magnetic fields.

## UDWT-Based Energy Detection Algorithm

**Figure 4:**
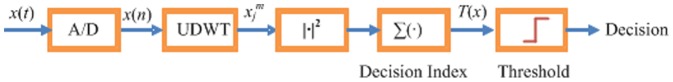
Block diagram of the detection system based on UDWT.

Consider the detection of a magnetic anomaly signal with unknown velocity ***v***, CPA distance *R*_0_ and magnetic moment ***M***, an energy detector based on UDWT is presented in this paper, and its block diagram is shown in Fig. 4.

Then the problem that whether the target magnetic anomaly signal exists in the output of the GMI sensor can be posed as a binary hypothesis as follows: 
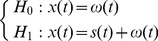
(4)here *x*(*t*) denotes the output signal of the GMI magnetic sensor, *s*(*t*) is the magnetic anomaly signal, while *ω*(*t*) is the 1/*f* background noise. Apparently, hypothesis *H*_0_ denotes only background noise exits in the measurement signal *x*(*t*), while hypothesis *H*_1_ indicates there are both the target signal and noise [17]–[19].

Wornell [6], [20] points out that the orthonormal wavelet decomposition can play a role of a Karhunen-Loève-type expansion to the 1/*f*-type signal by its decorrelation abilities. In other words, the wavelet coefficients *x_j_^m^* can approximate to be independent and considered as zero-mean normally distributed random variable. Thus, the two hypotheses given above can be re-expressed as: 
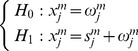
(5)where *x_j_^m^*, *ω_j_^m^* and *s_j_^m^* represent the wavelet coefficients of *x*(*t*), *ω*(*t*) and *s*(*t*) at *m*th scale, respectively, and subscript *j* denotes the *j*th wavelet coefficient. Assuming 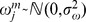, then the expression for the mean under *H*_1_ becomes:
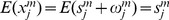
(6)and the variance is derived as follows:
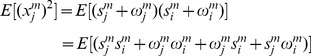
(7)


On account of the *ω_j_^m^* and *s_j_^m^* are uncorrelated, the last two terms are zero and the first two terms are non-zero only when *i* = *j*, then the above expression simplifies to:
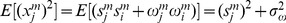
(8)


Based on the mentions above, it can be seen that under *H*_1_, the variance of the distribution is the same as under *H*_0_, but with a mean given by Eq. (6). In other words, the hypothesis can be re-written as:



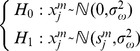
(9)


Based on Neyman-Person criterion and the energy detector [21], [22], then the detection statistic *T*(*x*) can be defined as:
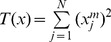
(10)


Here, *N* is the length of the signal *x*(*n*). Considering that the probability distribution shown in Eq. (9), the two hypotheses can be manipulated into the following form [13]: 
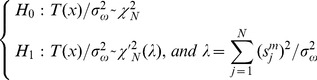
(11)here 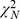 denotes an *N* degrees of freedom central chi-square *χ* ^2^ distribution, and 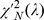 implies an *N* degrees of freedom non-central chi-square 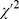 distribution, while its non-centrality parameter is *λ*. Assuming *γ* is the given detection threshold and *P*[•] is the probability function, then the probability of false alarm *P_FA_* can be given by:
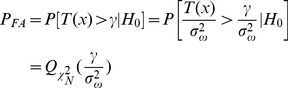
(12)


While the probability of detection *P_D_* can be expressed as:
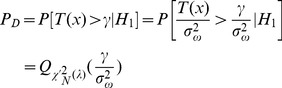
(13)


In addition, the variance 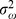 of the 1/*f* background noise is unknown and changed in practice, but it usually can be replaced by its maximum likelihood estimation (MLE) which is expressed as: 
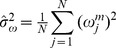
(14)


Based on Eq. (12), then the threshold *γ* can be obtained as: 
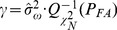
(15)


In other words, detection occurs when the detection index value exceeds the above threshold *γ*, as is depicted in Fig. 4.

## Results

In this section, some computer simulations have been performed to evaluate the presently proposed detection algorithm. The input signal *x*(*t*) that fed to the detector depicted in Fig. 4 was obtained by adding the noise samples with power spectral density of 1/*f* to a signal of a magnetic target moving with a constant velocity ***v***.

### 4.1 Selection of decomposition scale

Different decomposition scales have different computation, and the larger scale usually implies the more computation. Therefore, an appropriate scale is very critical to ensure that the proposed detector have a good real-time performance.

Considering the stronger relativity existing in the 1/*f* noise, the chosen wavelet function should be orthogonal which can simplify the calculation process, especially make the coefficients between inner and external scales have small relevance after wavelet decomposition [23]–[26]. Therefore, a set of quasi-orthogonal bi-orthogonal filters, which can be implemented by DSP chip [27], is selected as the quadrature mirror filters in this paper, and their coefficients are shown in Table 1 [23]. Here, *H*(*k*) and *G*(*k*) (here *k* = −11, −10,···, 10, 11) denote the decomposition low-pass and high-pass filter mentioned in Fig. 2, respectively.

**Table 1:** Coefficients of quasi-orthogonal bi-orthogonal filters (values×√2).

*k*	*H(k)*	*G(k)*	*k*	*H(k)*	*G(k)*
			0	0.561285256870	0.560116167736
**−1**	0.286503335274	−0.296144908701	1	0.302983571773	−0.296144908701
**−2**	−0.043220763560	−0.047005100329	2	−0.050770140755	−0.047005100329
**−3**	−0.046507764479	0.055220135661	3	−0.058196250762	0.055220135661
**−4**	0.016583560479	0.021983637555	4	0.024434094321	0.021983637555
**−5**	0.005503126709	−0.010536373594	5	0.011229240962	−0.010536373594
**−6**	−0.002682418671	−0.005725661541	6	−0.006369601011	−0.005725661541
**−7**	0	0.001774953991	7	−0.001820458916	0.001774953991
**−8**	0	0.000736056355	8	0.000790205101	0.000736056355
**−9**	0	−0.000339274308	9	0.000329665175	−0.000339274308
**−10**	0	−0.000047015908	10	0.000050192775	−0.000047015908
**−11**	0	0.000025466950	11	−0.000024465734	0.000025466950

**Figure 5:**
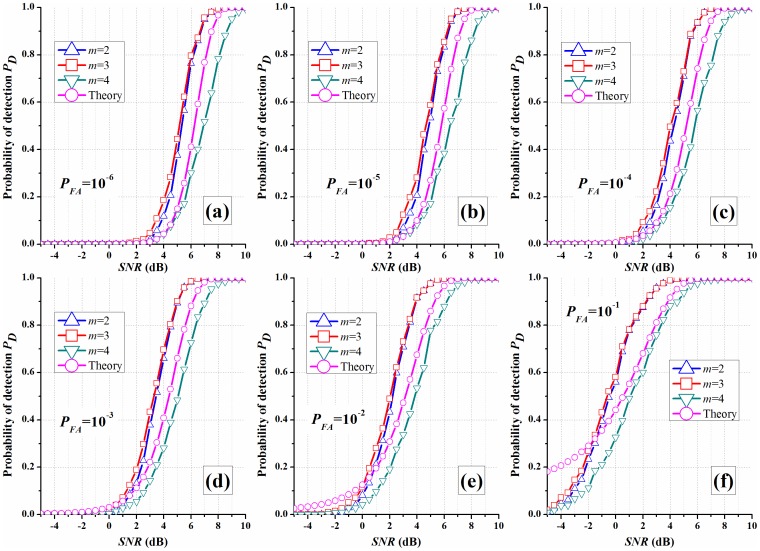
The probability of detection *P_D_* versus signal-to-noise ratio (*SNR*) with wavelet decomposition scale *m* and various probability of false alarm *P_FA_*: (a) *P_FA_* = 10^−6^; (b) *P_FA_* = 10^−5^; (c) *P_FA_* = 10^−4^; (d) *P_FA_* = 10^−3^; (e) *P_FA_* = 10^−2^; (f) *P_FA_* = 10^−1^.

In order to select an appropriate scale *m*, some simulations about the probability of detection *P_D_* with different wavelet decomposition scale *m* and given probability of false alarm *P_FA_* are evaluated, which of the results are shown in Fig. 5. At the same time, the probability of detection *P_D_* in theory with the corresponding probability *P_FA_* in the Gaussian white background noise also given out [17]. Here, the 1/*f* noises are generated based on the fractional Brown mention [24], [25], and the magnetic anomaly signals are simulated based on Eq. (2). The length *N* of the simulative input signal *x*(*n*) is 1024, while the length *N_S_* of magnetic anomaly signal *s*(*n*) is about 51. In addition, the spectral parameter *α* of simulative 1/*f* noise is 1.0, and each Monte Carlo simulation is repeated at least 1000 trials.

As illustrated in Fig. 5, it can be seen that the performance of the energy detector based on three-level UDWT decomposition is not only better than that of the detectors based on two-level and four-level UDWT decomposition with different probability of false alarm *P_FA_*, but also better than that in theory at the background of Gaussian white noise. Accordingly, the scale *m* is selected to 3 in this paper.

### 4.2 Detection performance

In this section, the receiver operating characteristics (ROC) with various spectral parameters *α* of our proposed detector would be obtained by utilized Monte Carlo simulations, and each simulation consisted of at least 1000 independent trials. As the same as the above section, the length *N* of the simulative input signal *x*(*n*) is 1024, and the length *N_S_* of magnetic anomaly signal *s*(*n*) is about 51, while *SNR* is 0 dB.

**Figure 6:**
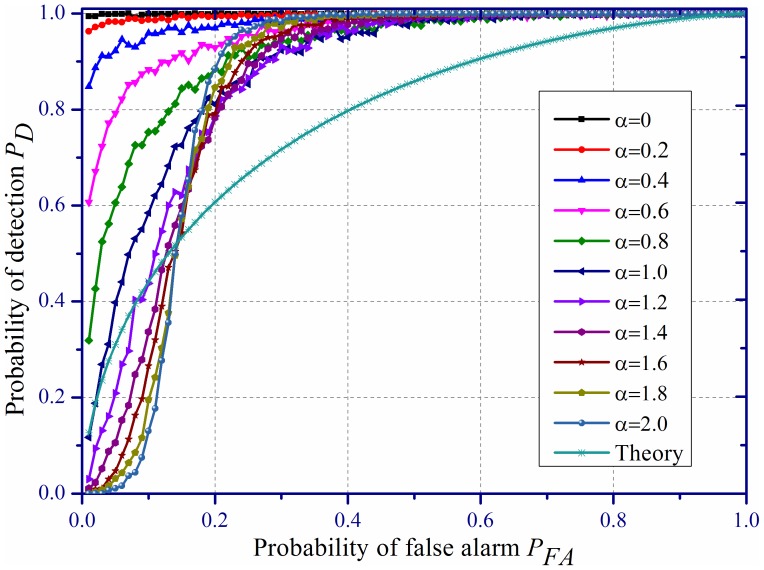
ROC curves of our proposed detector with different spectral parameters *α* when *SNR* = 0dB.

As illustrated in Fig. 6, it can be achieved that: (1) When the spectral parameter *α* is less than 1.0, the simulation detection performance of our proposed energy detector based on UDWT is better than that of the convention energy detector utilized in Gaussian white noise background [17]; (2) While the spectral parameter *α* is larger than 1.0, the detection performance of our detector is also better than that of the convention energy detector utilized in Gaussian white noise when the probability of false alarm *P_FA_* is larger than 0.15. These are may profited from the wavelet transform can effectively improve the output *SNR* [28], [29], i.e. the *SNR* of the coefficients *x_j_^m^* shown in Fig. 4.

### 4.3 Experiment results

**Figure 7:**
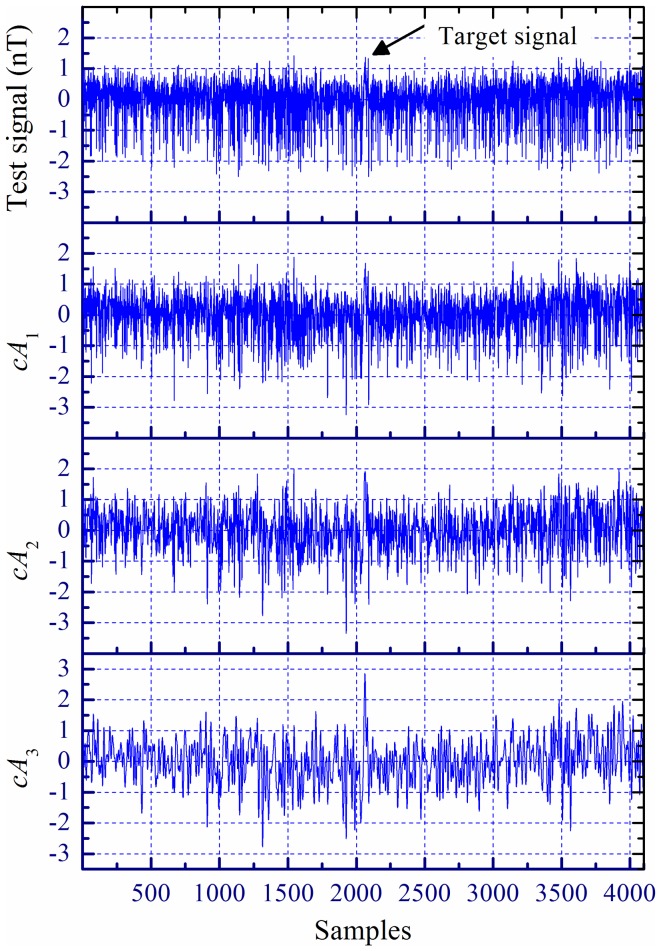
A magnetic target signal contaminated by real background noise: (a) Test signal; (b)–(d) First-, second-, and third-level approximation coefficients, respectively.

A test signal was produced by a moving magnetic target on a background of a real magnetic noise at a sampling rate of 1.0 kHz, which is shown in Fig. 7(a). Fig. 7(b)–(d) denote the first-, second- and third-level approximation coefficients (*cA*_1_, *cA*_2_, and *cA*_3_) of three-level UDWT decomposition, respectively. It is noticeable that the coefficients *cA_j_* (*j* = 1, 2, 3) are of length *N*, which is the same as the length of input signal *x*(*n*), instead of *N*/2 as in the DWT case.

As illustrated in Fig. 7(a), the test target signal is difficultly distinguish from the real background noise, while the operation become easy based on the coefficients *cA*_3_ shown in Fig. 7(d). Apparently, the output *SNR* is improved by the operation of UDWT. Although the value of spectral parameter *α* usually is an unknown priori in the real world magnetic noise, which even may not be exactly constant or somewhat vary with frequency [4], the detector based on UDWT employing a fixed set of quasi-orthogonal bi-orthogonal filters may handle it.

## Discussion and Conclusions

MAD is one of the most important geophysical techniques for detection and localization of the obscured ferromagnetic targets. Recently, MAD methods presented in the literatures can be divided into target-based method and noise-based method. However, the fore category is based on some priori assumptions of the magnetic anomaly target signal, such as the characteristic time *τ*. The later one needs know the magnetic background noise probability. In addition, many methods can be only utilized in the case of Gaussian white noise. In this study, we aimed at developing an energy detector based on UDWT to detect the magnetic anomaly signal not only contaminated by Gaussian white noise, but also by 1/*f* noise. In fact, after wavelet transforming by employing a set of quasi-orthogonal bi-orthogonal filters, not only the self-similarity and long-range correlation of the 1/*f* noise can be effectively removed, but also the signal-to-noise ratio (*SNR*) of the magnetic anomaly target signal can be improved. Besides, any priori information, such as the characteristic time *τ*, magnetic background noise probability or the spectral parameter *α*, is not required necessarily. Based on the simulation results of ROC with various spectral parameters *α* at the case of *SNR* is 0 dB, it can be seen that the detection performance of our proposed detector is better than that of the convention energy detector utilized in Gaussian white noise background, especially when the spectral parameter *α* is less than 1.0. Finally, for a real world magnetic noise, an experiment result shows that the operation based on UDWT is an effectiveness method for improve the *SNR* of the target signal. Our further research in magnetic anomaly detection will not only address the improvement of magnetic sensor and energy detector, but also focus on try other detection algorithms.
